# Fevers

**Published:** 1904-08-27

**Authors:** 


### Progress in Medicine and Surgery.

### FEVERS.

(*Continued from page* 362.)

Typhoid Fever (*continued*).—Hector MacKenzie [15] urges the importance of early diagnosis of perforation in typhoid fever. The medical attendant should foresee the possibility of this accident in every case, watch for it, and summon his previously selected surgeon on the first indication. Sudden abdominal pain, followed by changes in temperature, pulse, facies, and abdominal condition, is most significant. A. R. Parsons [16] deals with the same subject. Prognosis is discussed by R. E. Sedgwick.[17] The important question of *treatment* is handled by several authorities. Sir John Moore [18] emphasises the importance of pure air and good sanitation. He deprecates incautious purgation in early doubtful cases. Antipyretic drugs, antipyrin, phenacetin and others are contra-indicated: they depress the heart. The only safe antipyretic is cold water variously applied externally. Salicylate of quinine in 3-grain or 5-grain doses is given the first place as an intestinal antiseptic. Salicylate of bismuth is valuable and so also is turpentine or terebene 5 or 10 minims in capsule, or emulsified with spirits of nitrous ether and chloroform. It is antiseptic, antiputrefactive and astringent. For constipation before the eighth day $\frac{1}{2}$ to 3 grains of calomel is permissible. Later small repeated doses of castor-oil and glycerine or flipped banana (banana crushed and whipped like an egg) may be given. The latter is considered safe at any stage. A glycerine abdominal compress or a simple or turpentine enema are other remedies. Diarrhœa is met by revision of diet, elimination of broths, etc., and confinement to boiled milk and lime water, peptonised milk, or whey and egg water. Chalk mixture with the compound tincture of chloroform and morphia is useful. Repeated small hæmorrhages are much more ominous than a single copious bleeding. Absolute rest is essential, secured by withholding food or giving only whey and egg water, applying ice to the abdomen and exhibiting opium freely. Tannic acid, opium and turpentine combined with mucilage and peppermint-water is invariably effective in checking hæmorrhage. According to Murchison great caution must be exercised in advancing the diet as convalescence proceeds. A warning is given of the danger of routine treatment, especially in regard to alcoholic stimulants. H. P. Hawkins [19] points out that, apart from perforation, good treatment consists in attention to detail. Milk suitably diluted with the addition of glucose is the best diet. The nurse must exercise tact in employing flavouring agents, and varying the monotony by giving it as milk jelly made with isinglass and cream, or as milk soup flavoured with vegetables. Whey and egg-water may be partly substituted. Alcohol must be used strictly as a medicine. This writer admits no purgative from first to last, but relies on simple enemata every third day. Carbolic acid is recommended for meteorism and also as a disinfectant for the intestinal contents, not with any hope of killing off the bacilli. Acid carbol. ♏iii., Tinct. Chlorof. Co. ♏x., Tinct. Card. Co. ♏x, Syr. Aurantii ♏xl, Aq. Chlorof. ad 1℥ with iced water after food is found palatable. Cold water is here also the only antipyretic admitted. For hæmorrhage, rest and sufficient opium to prevent peristalsis are indicated, and in excessive cases infusion of normal saline solution. A week after a normal temperature has been reached and maintained, eggs and carbohydrates may be added to the diet. T. McCrae [20] describes the treatment as carried out under Osler in the wards of the Johns Hopkins Hospital. The diet during the febrile stage consists of milk and albumin water given alternately every two hours. Four ounces of milk with two of lime water alternates with four ounces of albumin water (one or two eggs being used) flavoured with lemon and sugar. Whey is often substituted for milk, as are koumiss and butter-milk, where milk is much disliked. The milk may also be boiled or peptonised. Tea, coffee, and ice cream are often permitted. Beef-tea and meat extracts are never given. Large quantities of water, even to 6 or 7 litres a day, are insisted upon as being extremely important. "More water" should be the motto in a typhoid ward, and "Too little food rather than too much." Baths are found to have an excellent effect on the nervous, circulatory, respiratory, and digestive systems, on the skin, temperature, and on the elimination of toxines. By the bath treatment, systematically carried out, probably five to seven patients are saved in every hundred. Objections can generally be overcome by tact and attention to detail. The patients are tubbed every three hours when the temperature is at or over 102·5° Fahr. Never are more than seven baths given in the 24 hours. Each lasts 15 or 20 minutes. The temperature of the water varies from 70° to 85°, according to the needs of the case. Constant rubbing whilst in the bath is essential. Shivering is of no importance. Unusual distress, cyanosis, or collapse, which are rare, indicate removal. Hæmorrhage, severe abdominal pain, phlebitis, cholecystitis, signs indicative of peritonitis or perforation, great prostration, and extremes of age are contra-indications. Rest, diet and baths constitute the treatment. Many patients get no medicine, most get not a drop of alcohol, which is only used for severe general conditions not because of the baths. Careful cleansing of the mouth, the use of simple enemata, with turpentine for tympanites, removal of milk if diarrhœa occurs, and avoidance of purgatives are other points mentioned. Strychnine and digitalis are used if indicated. During convalescence conservative methods prevail, and diet is only very gradually increased, broths, beef-tea, custard, jelly, etc, being very cautiously added. Calcium chloride gr. xv. three times daily is preferred to opium in cases of hæmorrhage wherever possible, as opium masks peritonitis and perforation. In suspicious cases of perforation exploration under cocaine is considered justifiable.

[15] Pract., Jan. [16] Dublin Jour. Med Sc., Feb. [17] Birm. Med. Rev, Feb. [18] Pract., Jan. [19] Ibid. [20] Ibid.

(*To be continued.*)

